# Sarcopenia and mortality risk in community-dwelling Brazilian older adults

**DOI:** 10.1038/s41598-022-22153-9

**Published:** 2022-10-20

**Authors:** Cristina Camargo Pereira, Valéria Pagotto, Cesar de Oliveira, Erika Aparecida Silveira

**Affiliations:** 1 Postgraduate Program in Health Sciences, Medical School, Federal University of Goiás (UFG), Goiania, Brazil; 2 Postgraduate Program in Nursing, Faculty of Nursing, Federal University of Goiás (UFG), Goiania, Brazil; 3 Department of Epidemiology and Public Health, University College London, London, UK

**Keywords:** Ageing, Risk factors

## Abstract

We estimated the impact of sarcopenia parameters on mortality risk and assessed its prevalence and associated factors in the older adults according to the European Working Group on Sarcopenia in Older People’s 2010 (EWGSOP1) and 2018 (EWGSOP2) criteria. This was a 10-year follow-up cohort study. Low muscle mass (MM) was defined as low skeletal muscle mass index (SMI) using dual-energy X-ray absorptiometry (DXA), and low calf circumference (CC). Cox regression and the Kaplan–Meier method were performed. The prevalence of sarcopenia and associated factors were influenced by the MM measurement method and diagnostic criteria used [6.8% (SMI and EWGSOP2), 12.8% (CC and EWGSOP2; and SMI and EWGSOP1) and 17.4% (CC and EWGSOP1)]. While a low BMI was associated with sarcopenia regardless of the sarcopenia definitions, diabetes, and high TGs were associated with sarcopenia only when using the EWGSOP1 criteria. Low SMI increased mortality risk (EWGSOP1: HR = 2.01, 95% CI 1.03–3.92; EWGSOP2: HR = 2.07, 95% CI 1.05–4.06). The prevalence of sarcopenia was higher according to EWGSOP1 than EWGSOP2. A low BMI, diabetes, and high TGs were associated with sarcopenia. A low SMI doubled the risk of mortality in community-dwelling older adults.

## Introduction

Sarcopenia was originally characterized by the loss of muscle mass associated with advancing age^1,2^. Recent definitions define sarcopenia as a reduction in mass, strength, and muscle function^3–9^. In 2010, the European Working Group on Sarcopenia in Older People (EWGSOP1) criteria recommended diagnosing sarcopenia based on a low muscle mass (MM) combined with low muscle strength and/or poor physical performance. A new consensus on sarcopenia was published, i.e., the EWGSOP2 criteria, wherein low muscle strength was defined as “probable sarcopenia” with low MM confirming the diagnosis. The new definition does not consider muscle function as a component of sarcopenia diagnosis but rather to classify the severity of the disease. Another difference between the two criteria was the modification of the diagnostic criteria to define both low MM and strength^4^.

Few studies in older adults applied both criteria to estimate the prevalence of sarcopenia. They found its prevalence to be significantly lower using the EWGSOP2 than the EWGSOP1^10–15^. Because the diagnosis of sarcopenia was reviewed by the EWGSOP2^4^, it is important to assess the impact of this new diagnostic criteria in clinical practice to understand the associated factors and mortality risk of sarcopenia in community-dwelling older adults.

Sarcopenia and its separate components have been associated with adverse health outcomes such as increased mortality risk^16–18,23^, diabetes^19,20,22^ and reduced weight^21^ later in life. Its diagnosis is highly relevant, both in research and clinical practice, although the evaluation of muscle mass is one of the major challenges^22,23^. The muscle mass measurement tools proposed by the EWGSOP1, such as Dual-energy X-ray absorptiometry (DXA), have limited clinical application. Conversely, the EWGSOP2 proposed the use of calf circumference (CC), as an alternate measure^4^.

The new recommendations of the EWGSOP2^4^ should be used to assess sarcopenia prevalence, its associated factors and mortality risk. They should also be compared with the previous diagnostic criteria. The new criteria changes are likely to result in different findings in the identification of cases in clinical practice and, consequently, in associated factors. Therefore, we aimed to assess the impact of sarcopenia and its parameters on mortality over a 10-year follow-up period in community-dwelling older adults. We also estimated the prevalence of sarcopenia and identified the factors associated with it using two methods for measuring MM and both EWGSOP1 and EWGSOP2 diagnostic criteria.

## Results

The study population consisted of 132 community-dwelling older adults (60.6% women) with a mean age of 70.0 ± 6.3 years. A positive gradient of increasing prevalence of sarcopenia with age was observed for all definitions evaluated. We highlight that the proportion of participants with sedentary physical activity change according to the different methods and definitions used. Older adults with low CC have a higher prevalence of sedentary lifestyle (EWGSOP1: 20%; EWGSOP2: 14%) than those with low skeletal muscle mass index (SMI) (EWGSOP1: 16%; EWGSOP2: 4%) in the definition of both criteria. The prevalence of sarcopenia was higher in underweight older adults ranging from 14.3% (using SMI and EWGSOP2) to 42.4% (using CC and EWGSOP1). Approximately one third (28%) of older adults with diabetes mellitus and more than half with hypertension (56.8%) had sarcopenia (using CC and EWGSOP1) (Table 1).

**Table 1:** Prevalence and factors associated with sarcopenia defined by the EWGSOP1 and EWGSOP2 criteria and two methods of measuring muscle mass (n = 132).

Variables	Total, n (%)	Prevalence of sarcopenia							
		EWGSOP1				EWGSOP2			
		Muscle mass measurement method							
		CC		SMI		CC		SMI	
		Yes, n (%)	p	Yes, n (%)	p	Yes, n (%)	p	Yes, n (%)	p
**Sex**									
Men	52 (39.4)	9 (17.3)	0.977	8 (15.4)	0.488	8 (15.4)	0.488	6 (11.5)	0.083
Women	80 (60.6)	14 (17.5)		9 (11.3)		9 (11.3)		3 (3.7)	
**Age groups**									
60–69	69 (52.3)	10 (14.5)	0.479	8 (11.6)	0.796	5 (7.3)	0.072	2 (2.9)	0.079
70–79	51 (38.6)	10 (19.6)		7 (13.7)		9 (17.6)		5 (9.8)	
80 and older	12 (9.1)	3 (25.0)		2 (16.7)		3 (25.0)		2 (16.7)	
**Skin colour**									
White	68 (51.5)	10 (14.7)	0.396	11 (17.2)	0.152	9 (14.1)	0.694	6 (9.4)	0.258
Not white	64 (48.5)	13 (20.3)		6 (8.8)		8 (17.8)		3 (4.4)	
**Schooling years**									
Illiterate	32 (26.2)	4 (12.5)	0.717	2 (6.3)	0.493	3 (9.4)	0.675	1 (3.3)	0.759
1–4 years	45 (36.9)	9 (20.0)		8 (17.8)		6 (13.3)		4 (8.9)	
5–8 years	30 (24.6)	7 (23.3)		5 (16.7)		6 (20.0)		3 (10.0)	
9 years or more	15 (12.3)	3 (20.0)		2 (13.3)		2 (13.3)		1 (6.7)	
Socioeconomic class*									
A/B/C	34 (26.4)	5 (14.7)	0.579	5 (14.7)	0.759	4 (11.7)	0.776	2 (5.9)	0.770
D/E	95 (7.6)	18 (18.9)		12 (12.6)		13 (13.7)		7 (7.4)	
**Living with a partner**									
No	76 (57.6)	11 (14.5)	0.298	10 (13.2)	0.911	10 (13.2)	0.911	6 (7.9)	0.568
Yes	56 (42.4)	12 (21.4)		7 (12.5)		7 (12.5)		3 (5.4)	
**Smoking status**									
Never	64 (48.5)	11 (17.2)	0.135	8 (12.5)	0.585	7 (10.9)	0.179	4 (6.3)	0.497
Current	14 (10.6)	5 (35.7)		3 (21.4)		4 (28.6)		2 (14.3)	
Ex-smoker	54 (40.9)	7 (13.0)		6 (11.1)		6 (11.1)		3 (5.6)	
**Alcohol consumption**									
No	103 (78.0)	19 (18.5)	0.392	14 (13.6)	0.459	14 (13.6)	0.459	8 (7.8)	0.371
Yes	29 (22.0)	4 (13.8)		3 (10.3)		3 (10.3)		1 (3.5)	
**Physical activity**									
Sedentary	107 (81.1)	5 (20.0)	0.706	15 (14.0)	0.419	4 (16.0)	0.605	1 (4.0)	0.535
Active	25 (18.9)	18 (16.8)		2 (8.0)		13 (12.2)		8 (7.5)	
**Daily consumption of fruits and vegetables**									
No	79 (59.8)	13 (16.5)	0.720	8 (15.1)	0.534	8 (15.1)	0.534	4 (7.5)	0.785
Yes	53 (40.2)	10 (18.8)		9 (11.4)		9 (11.4)		5 (6.3)	
**Comorbidities**									
Diabetes *mellitus*	37 (28.0)	2 (5.4)	**0.016**	1 (2.7)	**0.021**	2 (5.4)	0.090	1 (2.7)	0.224
Hypertension	75 (56.8)	13 (17.3)	0.975	12 (16.0)	0.219	9 (12.0)	0.730	6 (8.0)	0.537
**Nutritional status**									
Low weight**	56 (42.4)	20 (35.7)	** < 0.001**	14 (25.0)	**0.002**	15 (26.8)	** < 0.001**	8 (14.3)	**0.019**
Overweight	44 (33.3)	3 (6.8)		2 (4.5)		2 (4.5)		1 (2.3)	
Obese	32 (24.3)	-		1 (3.0)		-		-	
Cholesterol total, mean (DP)	201.99 (± 41.12)	197.17 (± 34.78)	0.538	199.12 (± 30.05)	0.758	195.47 (± 34.01)	0.486	199.44 (± 31.11)	0.848
HDL, mean (DP)	45.37 (± 12.36)	49.39 (± 15.93)	0.232	47.17 (± 16.35)	0.932	47.29 (± 12.88)	0.461	47.33 (± 11.61)	0.456
LDL, mean (DP)	124.24 (± 34.87)	123.13 (± 33.11)	0.867	124.47 (± 28.99)	0.977	122.41 (± 30.76)	0.817	125.78 (± 28.55)	0.892
TG, mean (DP)	149.85 (± 62.51)	123.22 (± 57.89)	**0.018**	137.65 (± 58.49)	0.566	128.59 (± 57.77)	0.100	132.0 (± 44.10)	0.524
Fasting glucose, mean (DP)	107.72 (± 38.48)	100.87 (± 38.87)	**0.044**	94.82 (± 28.93)	**0.021**	105.88 (± 44.28)	0.302	102.22 (± 38.69)	0.381

*EWGSOP* European Working Group on Sarcopenia in older people, *CC* calf circumference, *DXA* X-ray double absorption bone densitometry, *BMI* body mass index, *HDL* high density lipoprotein (mg/dL) (high density lipoprotein), *LDL* low density lipoproteins (mg/dL) (low density lipoprotein), *TG* triglycerides (mg/dL), *SD* standard deviation, *SMI* skeletal muscle mass index.

Significant values are in bold.

Statistical analysis: independent t tests for continuous variables with a normal distribution, and the Mann–Whitney U test for continuous data with an abnormal distribution; and Pearson chi-square test or Fisher exact test (with the expected cell count of < 5) for categorical variables.

*Class A (n = 1) and Class B (n = 2).

**Nutritional status: low weight (n = 5), eutrophic (n = 51).

### Prevalence of sarcopenia

Considering EWGSOP1, the prevalence of sarcopenia was 17.4% (CC) and 12.8% (skeletal muscle mass index [SMI]). Considering EWGSOP2, the prevalence was 12.8% (CC) and 6.8% (SMI). The prevalence of “pre-sarcopenia” was 28.3% (EWGSOP1), and the prevalence of “probable sarcopenia” was 34.4% (EWGSOP2).

### Factors associated with sarcopenia

Factors associated with sarcopenia according to the EWGSOP1 criteria using CC in the evaluation of low muscle mass were diabetes mellitus (DM) (p < 0.016), low body mass index (BMI) (p < 0.001), high triglycerides (TG’s) (< 0.018), and fasting glycemia (p < 0.044). Factors associated with sarcopenia according to the EWGSOP1 criteria using SMI were DM (p < 0.021), low BMI (p < 0.002), and fasting glycemia (p < 0.021). According to the EWGSOP2, the only factor associated with sarcopenia was a low BMI, when using either CC (p < 0.001) or SMI (p < 0.019) for the muscle mass evaluation (Table 1).

### Mortality risk: association with sarcopenia and its parameters

**Figure 1:**
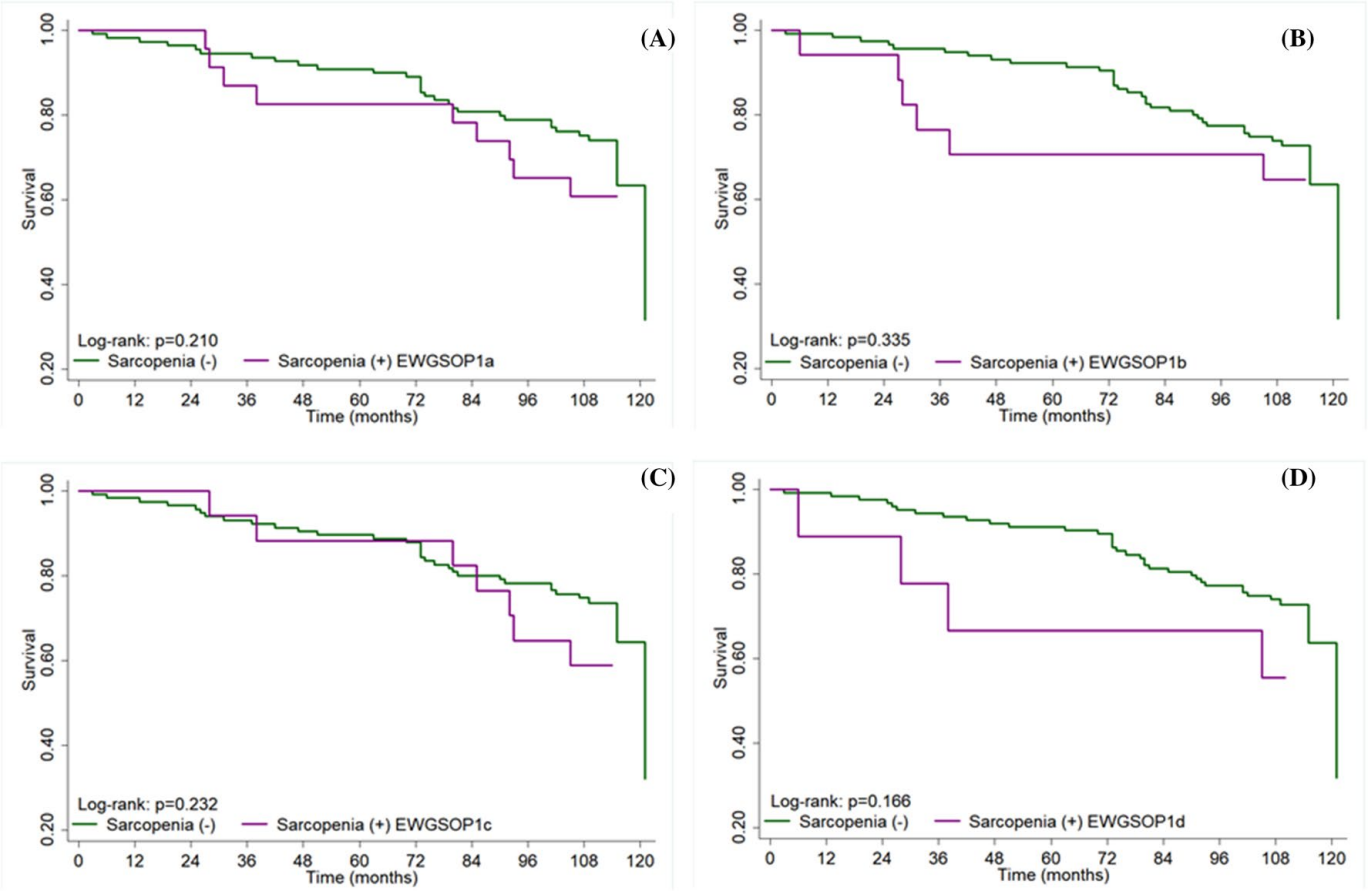
Survival curves according to sarcopenia status according to EWGSOP1 (**A**,**B**) and EWGSOP2 (**C**,**D**) criteria. *EWGSOP* European Working Group on Sarcopenia in Older People. (**A**) EWGSOP1^a^: Low Calf circumference + Low Handgrip strength. (**B**) EWGSOP1^b^: Low Skeletal muscle mass index + Low Handgrip strength. (**C**) EWGSOP2^c^: Low Handgrip strength + Low Calf circumference. (**D**) EWGSOP2^d^: Low Handgrip strength + Low Skeletal muscle mass index.

The proportion of deaths was significantly higher in older adults with “probable sarcopenia” (p < 0.043). Low muscle mass by CC was observed in 32.6% individuals (Table 2). Low SMI was associated with increased mortality risk, considering the two criteria (EWGSOP1: hazard ratio [HR] = 2.01, 95% confidence interval [CI] 1.03–3.92; EWGSOP2: HR = 2.07, 95% CI 1.05–4.06). Sarcopenia, low CC, and low Handgrip strength (HS) were not associated with mortality (Table 3). There was no multicollinearity between the independent variables (variance inflation factor [VIF] < 10.0) (Table S1). Results of the survival curves according to sarcopenia status by the EWGSOP1 and EWGSOP2 criteria showed no significant differences (Fig. 1).

**Table 2:** Prevalence of sarcopenia, pre-sarcopenia and probable sarcopenia by the EWGSOP1 and EWGSOP2 criteria, two muscle mass assessment methods (n = 132) and association with all-cause mortality (n = 39).

Variables	Prevalence, n (%)	All-cause mortality, n (%)	p
**CC**			
Normal	89 (67.4)	23 (25.8)	0.180
Low	43 (32.6)	16 (37.2)	
**Pre-sarcopenia**			
EWGSOP1	37 (28.3)	15 (40.5)	0.084
EWGSPO2	32 (24.2)	14 (43.8)	**0.043**
**Probable sarcopenia**			
EWGSOP1	71 (54.2)	19 (26.7)	0.538
EWGSOP2	45 (34.4)	11 (24.4)	0.405
**Sarcopenia**			
EWGSOP1^a^	23 (17.4)	9 (39.1)	0.268
EWGSOP1^b^	17 (12.8)	6 (35.3)	0.578
EWGSOP2^c^	17 (12.8)	7 (41.2)	0.260
EWGSOP2^d^	9 (6.8)	4 (44.4)	0.254

Statistical analysis: Chi-square test.

*CC* calf circumference, *EWGSOP* European Working Group on Sarcopenia in older people, *SD* standard deviation.

Significant values are in bold.

EWGSOP1^a^: Low Calf circumference + Low Handgrip strength.

EWGSOP1^b^: Low Skeletal muscle mass index + Low Handgrip strength.

EWGSOP2^c^: Low Handgrip strength + Low Calf circumference.

EWGSOP2^d^: Low Handgrip strength + Low Skeletal muscle mass index.

*Missing data for 1 female participant.

**Table 3:** Crude and adjusted cox regression analysis between the parameters of sarcopenia, by the EWGSOP1 and EWGSOP2 sarcopenia criteria, according to two muscle mass measuring methods and 10-year all-cause mortality risk.

Variables	EWGSOP1				EWGSOP2			
	Crude		Adjusted		Crude		Adjusted	
	HR (CI 95%)	p	HR (CI 95%)	p	HR (CI 95%)	p	HR (CI 95%)	p
Low CC	1.47 (0.78–2.80)	0.235	1.45 (0.74–2.85)	0.278				
Low SMI	**1.81 (1.00–3.47)**	**0.035**	**2.01 (1.03–3.92)**	**0.041**	**2.03 (1.05–3.93)**	**0.035**	**2.07 (1.05–4.06)**	**0.035**
Low HS	0.91 (0.48–1.74)	0.780	0.90 (0.46–1.78)	0.768	0.79 (0.39–1.61)	0.51	0.54 (0.26–1.14)	0.104
**Sarcopenia**								
CC	1.61 (0.76–3.40)	0.214	1.33 (0.58–3.03)	0.503	1.64 (0.72–3.75)	0.238	1.42 (0.59–3.42)	0.435
SMI	1.53 (0.64–3.67)	0.340	2.16 (0.86–5.39)	0.100	2.05 (0.73–5.79)	0.176	2.51 (0.86–7.26)	0.091

Adjusted for: age, sex, smoking, physical activity, and diabetes mellitus.

*HR* hazard ratio, *CI* confidence interval, *CC* calf circumference, *SMI* skeletal muscle mass index, *HS* handgrip strength, *EWGSOP* European Working Group on Sarcopenia in older people.

Significant values are in bold.

## Discussion

Our findings showed that the use of different diagnostic criteria for sarcopenia and different methods of measuring muscle mass (MM) resulted in variability of estimates of the sarcopenia prevalence and its associated factors in community-dwelling older adults. Overall, the risk factors identified were diabetes, low BMI, serum glycemia alterations, and high triglycerides. We would like to highlight that low SMI indicated a two-fold higher all-cause mortality risk than low calf circumference (CC). The proportion of individuals with obesity in the study sample may account for this finding. Compared with individuals in the lowest tertile, participants in the highest CC tertile had a higher prevalence of higher BMI values (Table S2).

To the best of our knowledge, this is the first study to compare the impact of sarcopenia and low muscle mass in community-dwelling older adults in a 10-year follow-up by applying the two methods of measuring muscle mass (CC and SMI) and the diagnostic criteria of the EWGSOP1 and EWGSOP2.

In this study, the overall prevalence of sarcopenia was 6.8–17.4%. This figure corroborates previous studies that reported a prevalence of 5–13% at 60–70 years of age and 11–50% at ≥ 80 years of age^7,24^. A recent meta-analysis reported a prevalence of sarcopenia ranging from 8.6 to 36.5% in community-dwelling older adults^25^. The variability in prevalence observed in the present study is due to the different diagnostic criteria for sarcopenia and the two methods of measuring muscle mass applied, which is in accordance with some of the previous studies^26,27^.

We observed that the prevalence of sarcopenia, regardless of the muscle mass measurement method (CC or SMI), was higher applying the EWGSOP1 criteria in relation to the EWGSOP2. Previous studies on the prevalence of sarcopenia comparing the two criteria found similar results to ours^12–15^. A study using CC observed the prevalence of sarcopenia to be more than double for the EWGSOP1 (8.8%) than the EWGSOP2 (3.4%) criteria^28^.

The differences in prevalence between EWGSOP1 and EWGSOP2 criteria can be explained by the change in algorithm and cutoff points. According to the new algorithm (EWGSOP2), older adults with reduced muscle mass but with normal strength and function are classified without sarcopenia^4^. A previous study stated that the use of EWGSOP2 may not be appropriate as a diagnostic tool for sarcopenia in high prevalence settings such as care homes and hospitals^29^. In the present study, a 0.5 T-score (EWGSOP1: − 2.0 T-scores and EWGSOP2: − 2.5 T-scores) difference in cut-offs resulted in relatively large differences in the prevalence between these individual criteria, which translated to large differences in sarcopenia and “pre-sarcopenia” or “probable sarcopenia” (Table 2).

The choice of tools for diagnosing sarcopenia depends on the characteristics of the individual, access to technical resources, and the main objective, i.e., monitoring the progression, rehabilitation and recovery^4,30^.

In the present study, a BMI value lower than 25.0 kg/m^2^ was a risk factor associated with sarcopenia considering the two diagnostic criteria and the two methods of measuring muscle mass (CC and SMI). Our results confirm the conclusions of other similar studies^31–36^. A cohort study with community-dwelling older adults showed that a BMI < 18.5 kg/m^2^ was associated with a higher prevalence of sarcopenia^37^. A similar association was observed in another study with a BMI < 22 kg/m^2^ with institutionalized individuals^35^. Being overweight has been associated with improved survival, occasionally called the “obesity paradox”^38^.

Based on the EWGSOP1 criteria, we found that the risk factors associated with sarcopenia were diabetes and altered serum levels of glycemia and triglycerides. These results confirmed findings from previous studies^39–42^. Although the association between diabetes and the prevalence of sarcopenia is not fully understood, several mechanisms have been proposed for the acceleration of sarcopenia in older diabetic adults^39^. Insulin resistance is one of the mechanisms involved in the development of sarcopenia^43^. The loss of muscle mass contributes to impaired insulin metabolism, since muscle tissue is a target organ for insulin actions^44^.

Previous evidence suggested that reduced skeletal muscle mass increases the risk of developing dyslipidemia^37,45–47^. The infiltration of cholesterol in muscle tissue, which may be caused by elevated plasmatic triglycerides levels, may contribute to increased oxidative stress on muscles^48^ and, consequently, muscle damage^49,50^.

In this study, low skeletal muscle mass index (SMI) was associated with increased all-cause 10-year mortality risk. Our results are in agreement with a Chinese study in centenarian women in which SMI was a predictor of mortality^40^. Previous studies have shown that the evaluation of muscle mass alone is not enough to predict mortality^51,52^, and yet muscle strength is a better predictor of adverse outcomes such as mortality, than muscle mass^53–55^. However, in the present study, muscle strength, i.e., handgrip strength was not associated with an increased mortality risk. However, low SMI showed a two-fold increase in mortality risk over the 10-year follow-up period in community-dwelling older adults. Our finding that low HS in the absence of low SMI did not increase the risk of all-cause mortality may cause controversy when compared with previous studies. The mechanisms that explain the association of low muscle strength with increased risk of mortality in community-dwelling older adults are not well understood^56^. Studies are needed to verify whether the association between low muscle strength and mortality is direct or whether muscle strength is a marker of other factors underlying mortality^57^. Results from previous studies are similar to ours, in which they showed that initial grip strength was not associated with mortality^56,57^. This study presents baseline muscle strength results; therefore, it is believed that for this reason muscle strength was not associated with mortality. A clinical trial study with a senescent population of mice showed that loss of muscle quality preceded loss of absolute function because of maintaining larger and poorer quality muscles, which increases the metabolic demand to maintain larger muscles, and exacerbation of catabolism age-related muscle growth in older muscles if the metabolic energy needs of skeletal muscles are not met^58^.

Although this study showed a decreased survival rate in sarcopenic participants after a long-term follow-up, this finding was not significant. Previous studies presented divergent results in relation to a lower probability of survival in sarcopenic patients^18,23,59^. These conflicting findings can be explained by the age of participants, older individuals than in our study, shorter follow-up times, use of different muscle mass measurement methods such as bioelectrical impedance analysis (BIA) and the use of cutoff points for diagnosis of low muscle mass (CC < 31 cm) different from our study. A longitudinal study with methodological characteristics similar to ours regarding its sample size, age and use of DXA to measure muscle mass, observed similar results as those in the present study, i.e., sarcopenia was not associated with mortality in older women^59^.

As a potential limitation of this study, we could mention small number of participants since the DXA test was performed in a sub-sample of the study population. However, some strengths of this analysis were its long follow-up period allowing us to assess the impact of sarcopenia parameters on mortality risk, the inclusion of community-dwelling older adults with a wide age range (60–98 years), and the use of two methods to assess muscle mass, i.e., the DXA and CC that has applicability in clinical practice.

Future research should focus on exploring the mechanisms underlying the association between low muscle mass and mortality risk. The use of muscle mass assessment either by DXA or a more accessible technique such as CC is suggested in routine clinical practice, to reduce the occurrence of sarcopenia and to develop preventive intervention. Older adults with low BMI, elevated blood glucose and triglyceride levels should be under greater surveillance with enrollment in intervention programs to preserve muscle mass. Muscle mass reduction should be considered as one of the potential triggers of geriatric syndromes such as sarcopenia and mortality.

In summary, this study showed that the estimation of the prevalence of sarcopenia, associated factors and its impact on mortality risk depends on the criteria adopted and method of measuring muscle mass. The prevalence of sarcopenia was higher using the EWGSOP1 criteria than the EWGSOP2 one and when using CC instead of SMI. Low BMI was a risk factor for sarcopenia regardless of the definitions used. Using the EWGSOP1 criteria, we found that the presence of diabetes and changes in serum glycemia and triglyceride levels were also associated with the development of sarcopenia. Low SMI doubled the all-cause mortality risk in the 10-year follow-up in community-dwelling older adults. However, low muscle strength and low CC did not impact on mortality risk.

## Methods

### Study population

The Goiânia Ageing Project is a prospective cohort involving 418 community-dwelling older adults living in Goiania city, Midwestern Brazil, which started in 2008. In the first wave of the cohort (2009) biochemical tests and body composition assessments were performed on 132 older adults^60–66^. The sample selection was probabilistic and stratified according to the health regions linked to the health districts used in the organization and management of the Brazilian Unified Health System (SUS) of the municipality. This analysis included only those who participated in 2009^62^. The ethical review committee at the Medical School, Federal University of Goias, Goiania, Brazil (2,500,044/2018) approved this study. All participants provided an informed written consent. This study was conducted in accordance with the declaration of Helsinki^67^.

### Data collection procedures

Data collection was conducted in a specialized diagnostic imaging clinic for DXA analysis and blood collection. The inclusion criteria to perform these tests were fasting for at least 4 h, no alcohol or caffeine consumption in the last 24 h, no physical activity performed in the last 12 h, emptying the bladder 30 min before the data collection, and no use of diuretics in the last 24 h. The blood samples were collected after 12-h fasting.

### Muscle mass (MM) assessment

MM was assessed through DXA (software version 7.52.002, GE-Lunar DPX-MD PLUS) and calf circumference (CC). Using the arms and legs MM estimated by DXA, skeletal muscle mass index (SMI) was calculated as follows: appendicular skeletal muscle mass of the arms + appendicular skeletal muscle mass of the legs/height^2^^1^. A low MM was defined according to EWGSOP1 as SMI ≤ 7.26 kg/m^2^ for men and ≤ 5.5 kg/m^2^ for women and by EWGSOP2 as SMI ≤ 7.0 kg/m^2^ for men and ≤ 5.5 kg/m^2^ for women.

CC was measured on the left leg with an inelastic tape measure (CESCORF), in its most protruding part, with the participant in an upright position^68^. The anthropometric evaluation was performed by qualified nutritionists according to the Habicht’s technique^69^. A CC of < 34 cm for men and < 33 cm for women were used as indicators of low MM, and these cutoff points were previously validated in the present study sample^63^.

### Muscle strength assessment

Handgrip strength (HS) was measured using a manual hydraulic dynamometer (JAMAR). The test was performed with the individual sitting on a chair, upright vertical back without arm support, elbow flexed at 90° and forearm in a neutral position. Three HS measurements for the dominant hand were obtained and the highest value was used. According to the EWGSOP1, a HS < 30 kg for men and < 20 kg for women, and the EWGSOP2, a HS < 27 kg for men and < 16 kg for women, were indicative of low muscle strength.

### Sarcopenia assessment

Considering the criteria of the EWGSOP1, the EWGSOP2, and the evaluation of muscle mass by SMI and CC, sarcopenia was defined as: (1) EWGSOP1 A: Low CC + Low HS; (2) EWGSOP1 B: Low SMI + Low HS; (3) EWGSOP2 A: Low HS + Low CC; and (4) EWGSOP2 B: Low HS + Low SMI.

### Mortality ascertainment

All-cause mortality registers in the last 10 years (July 2009 to March 2019) were obtained through the Brazilian Mortality Information System (SIM) for the Municipal Health Secretariat (SMS).

### Sociodemographic characteristics, lifestyle and health conditions

The following covariates were included: sociodemographic (age, gender, ethnicity, level of education, socioeconomic class, living with a partner), lifestyle (smoking status, of alcohol consumption, physical activity level, daily consumption of fruits and vegetables), comorbidities (diabetes [DM] and hypertension), BMI (weight in kg/height in m^2^) categorized according to the World Health Organization cutoff points^70^, and biochemical tests (total cholesterol, HDL-cholesterol, LDL-cholesterol, triglycerides [TG], and fasting glycemia in mg/dL).

DM was diagnosed when the fasting glycemia level was ≥ 126 mg/dL and/or when hypoglycemic drugs were used^71^. Hypertension was diagnosed when the systolic pressure ≥ 140 mm Hg and the diastolic pressure ≥ 90 mm Hg and/or when hypotensive drugs were used^72^.

### Statistical analysis

The prevalence of sarcopenia and its associated factors were estimated according to the four definitions of sarcopenia used in this study. Their association with sociodemographic variables, lifestyle, and health conditions including biochemical tests were assessed.

To analyze the factors associated with the prevalence of sarcopenia we used independent t tests for continuous variables with a normal distribution, and the Mann–Whitney U test for continuous data with an abnormal distribution; and Pearson chi-square test or Fisher exact test (with the expected cell count of < 5) for categorical variables. The association analysis between sarcopenia, pre-sarcopenia and probable sarcopenia with all-cause mortality was performed using the chi-square test.

Unadjusted and fully adjusted Cox regression analyses were performed to estimate the association between low muscle mass (CC and SMI), low hand grip strength, sarcopenia, and 10-year mortality risk. Age, gender, smoking status, physical activity, and DM were included as covariates.

Kaplan–Meier survival curves were plotted, and the comparison between the groups with and without sarcopenia was performed using the log-rank test. For all tests, a 5% significance level was considered. Statistical analyses were performed using the Stata/SE version 12.0.

## Supplementary Information

Supplementary Information.

## Data Availability

The datasets generated during and/or analysed during the current study are available from the corresponding author on reasonable request.

## References

[1] Baumgartner RN (1998). Epidemiology of sarcopenia among the elderly in New Mexico. Am. J. Epidemiol..

[2] Rosenberg IH (1989). Summary comment. Am. J. Clin. Nutr..

[3] Cruz-Jentoft AJ (2010). Sarcopenia: European consensus on definition and diagnosis: Report of the European Working Group on Sarcopenia in older people. Age Ageing..

[4] Cruz-Jentoft AJ (2019). Sarcopenia: Revised European consensus on definition and diagnosis. Age Ageing..

[5] Muscaritoli M (2010). Consensus definition of sarcopenia, cachexia and pre-cachexia: Joint document elaborated by Special Interest Groups (SIG) “cachexia-anorexia in chronic wasting diseases” and “nutrition in geriatrics”. Clin. Nutr..

[6] Morley JE (2011). Sarcopenia with limited mobility: An international consensus. J. Am. Med. Dir Assoc..

[7] Fielding, R. A. *et al*. Sarcopenia: an undiagnosed condition in older adults. Current consensus definition: Prevalence, etiology, and consequences. International working group on Sarcopenia. *J. Am. Med. Dir. Assoc*. **2**(4), 249–56 (2011). 10.1016/j.jamda.2011.01.003 PMC3377163 21527165

[8] Chen LK (2014). Sarcopenia in Asia: Consensus report of the Asian working group for sarcopenia. J. Am. Med. Dir. Assoc..

[9] Studenski AS (2014). The FNIH sarcopenia project: Rationale, study description, conference recommendations, and final estimates. Gerontol. A Biol. Sci. Med. Sci..

[10] Zhuang CL (2019). EWGSOP2 versus EWGSOP1 for sarcopenia to predict prognosis in patients with gastric cancer after radical gastrectomy: Analysis from a large-scale prospective study. Clin. Nutr..

[11] Locquet M, Beaudart C, Petermans J, Reginster JY, Bruyère O (2019). EWGSOP2 versus EWGSOP1: Impact on the prevalence of Sarcopenia and its major health consequences. J. Am. Med. Dir. Assoc..

[12] Reiss J (2019). Consequences of applying the new EWGSOP2 guideline instead of the former EWGSOP guideline for sarcopenia case finding in older patients. Age Ageing..

[13] Shafiee G (2020). Comparison of EWGSOP-1and EWGSOP-2 diagnostic criteria on prevalence of and risk factors for sarcopenia among Iranian older people: The Bushehr Elderly Health (BEH) program. J. Diabetes Metab. Disord..

[14] Yang L (2020). Comparison of revised EWGSOP criteria and four other diagnostic criteria of sarcopenia in Chinese community-dwelling elderly residents. Exp. Gerontol..

[15] Savas S, Taşkıran E, Sarac FZ, Akcicek F (2019). A cross-sectional study on sarcopenia using EWGSOP1 and EWGSOP2 criteria with regional thresholds and different adjustments in a specific geriatric outpatient clinic. Eur. Geriatr. Med..

[16] Alexandre TDS, Duarte YADO, Santos JLF, Wong R, Lebrao ML (2014). Sarcopenia according to the European Working Group on Sarcopenia in Older People (EWGSOP) versus dynapenia as a risk factor for mortality in the elderly. J. Nutr. Health Aging..

[17] Studenski S (2011). Gait speed and survival in older adults. J. Am. Med. Assoc..

[18] Landi F (2012). Sarcopenia and mortality among older nursing home residents. J. Am. Med. Dir. Assoc..

[19] Umegaki H (2015). Sarcopenia and diabetes: Hyperglycemia is a risk factor for age-associated muscle mass and functional reduction. J. Diabetes Investig..

[20] Cleasby ME, Jamieson PM, Atherton PJ (2016). Insulin resistance and sarcopenia: Mechanistic links between common co-morbidities. J. Endocrinol..

[21] Alexandre TDS, Duarte YADO, Santos JLF, Wong R, Lebrão ML (2014). Prevalence and associated factors of sarcopenia among elderly in Brazil: Findings from the sabe study. J. Nutr. Heal Aging..

[22] Dupuy C (2015). Searching for a relevant definition of sarcopenia: Results from the cross-sectional EPIDOS study. J. Cachexia Sarcopenia Muscle..

[23] Morley JE, Malmstrom TK (2014). Can sarcopenia be diagnosed without measurements?. Eur. Geriatr. Med..

[24] von Haehling S, Morley JE, Anker SD (2010). An overview of sarcopenia: Facts and numbers on prevalence and clinical impact. J. Cachexia Sarcopenia Muscle..

[25] Liu P, Hao Q, Hai S, Wang H, Cao L, Dong B (2017). Sarcopenia as a predictor of all-cause mortality among community-dwelling older people: A systematic review and meta-analysis. Maturitas.

[26] Shafiee G, Keshtkar A, Soltani A, Ahadi Z, Larijani B, Heshmat R (2017). Prevalence of sarcopenia in the world: A systematic review and meta-analysis of general population studies. J. Diabetes Metab. Disord..

[27] Beaudart C, Reginster JY, Slomian J, Buckinx F, Locquet M, Bruyère O (2014). Prevalence of Sarcopenia: The impact of different diagnostic cut-off limits. J. Musculoskelet. Neuronal. Interact..

[28] Barbosa-Silva TG, Bielemann RM, Gonzalez MC, Menezes AM (2016). Prevalence of sarcopenia among community-dwelling elderly of a medium-sized South American city: Results of the COMO VAI? study. J. Cachexia Sarcopenia Muscle..

[29] Cruz-Jentoft, A. J. *et al.* Prevalence of and interventions for sarcopenia in ageing adults: A systematic review. Report of the International Sarcopenia Initiative (EWGSOP and IWGS). *Age Ageing.* **43**(6), 748–59 (2014). 10.1093/ageing/afu115 PMC4204661 25241753

[30] Mijnarends, D.M. *et al*. Validity and reliability of tools to measure muscle mass, strength, and physical performance in community-dwelling older people: A systematic review. *J. Am. Med. Dir. Assoc.* **14**(3), 170–178 (2013). 10.1016/j.jamda.2012.10.009 23276432

[31] Smoliner C, Sieber CC, Wirth R (2014). Prevalence of sarcopenia in geriatric hospitalized patients. J. Am. Med. Dir. Assoc..

[32] Gariballa S, Alessa A (2013). Sarcopenia: Prevalence and prognostic significance in hospitalized patients. Clin. Nutr..

[33] Landi, F., *et al*. Prevalence and risk factors of sarcopenia among nursing home older residents*. J. Gerontol. A Biol. Sci. Med. Sci*. **6**7(1), 48–55 (2012). 10.1093/gerona/glr035 21393423

[34] Bastiaanse LP, Hilgenkamp TIM, Echteld MA, Evenhuis HM (2012). Prevalence and associated factors of sarcopenia in older adults with intellectual disabilities. Res. Dev. Disabil..

[35] Bravo-José P, Moreno E, Espert M, Romeu M, Martínez P, Navarro C (2018). Prevalence of sarcopenia and associated factors in institutionalised older adult patients. Clin. Nutr. ESPEN..

[36] Su Y, Hirayama K, Han T, Izutsu M, Yuki M (2019). Sarcopenia prevalence and risk factors among Japanese community dwelling older adults living in a snow-covered city according to EWGSOP2. J. Clin. Med..

[37] Khongsri N, Tongsuntud S, Limampai P, Kuptniratsaikul V (2016). The prevalence of sarcopenia and related factors in a community-dwelling elders Thai population. Osteoporos Sarcopenia..

[38] Bischoff SC, Boirie Y, Cederholm T, Chourdakis M, Cuerda C, Delzenne NM (2017). Towards a multidisciplinary approach to understand and manage obesity and related diseases. Clin. Nutr..

[39] Kim H (2015). Incidence and predictors of sarcopenia onset in community-dwelling elderly Japanese women: 4-Year follow-up study. J. Am. Med. Dir. Assoc..

[40] Wang T (2016). Type 2 diabetes mellitus is associated with increased risks of sarcopenia and pre-sarcopenia in Chinese elderly. Sci. Rep..

[41] Sobestiansky S, Michaelsson K, Cederholm T (2019). Sarcopenia prevalence and associations with mortality and hospitalisation by various sarcopenia definitions in 85–89 year old community-dwelling men: A report from the ULSAM study. BMC Geriatr..

[42] Umegaki, H. Sarcopenia and frailty in older patients with diabetes mellitus. Geriatrics and Gerontology International. *Geriatr. Gerontol. Int*. **16**(3), 293–9 (2016). 10.1111/ggi.12688 26799937

[43] Petersen MC, Shulman GI (2018). Mechanisms of insulin action and insulin resistance. Physiol. Rev..

[44] Landi F (2019). Muscle loss: The new malnutrition challenge in clinical practice. Clin. Nutr..

[45] Scott D (2016). Associations of low muscle mass and the metabolic syndrome in Caucasian and Asian middle-aged and older adults. J. Nutr. Heal. Aging..

[46] Baek, S. J. *et al*. Sarcopenia and sarcopenic obesity and their association with dyslipidemia in Korean elderly men: The 2008–2010 Korea National Health and Nutrition Examination Survey. *J. Endocrinol. Invest*. **37**(3), 247–60 (2014). 10.1007/s40618-013-0011-3 24615361

[47] Atlantis E, Martin SA, Haren MT, Taylor AW, Wittert GA (2009). Inverse associations between muscle mass, strength, and the metabolic syndrome. Metabolism.

[48] Sellers, S. L. *et al*. Increased nonHDL cholesterol levels cause muscle wasting and ambulatory dysfunction in the mouse model of LGMD2B. *J. Lipid Res*. **59**(2), 261–272 (2018). 10.1194/jlr.M079459 PMC5794421 29175948

[49] Whitehead NP, Yeung EW, Allen DG (2006). Muscle damage in mdx (dystrophic) mice: Role of calcium and reactive oxygen species. Clin. Exp. Pharmacol. Physiol..

[50] Terrill JR, Radley-Crabb HG, Iwasaki T, Lemckert FA, Arthur PG, Grounds MD (2013). Oxidative stress and pathology in muscular dystrophies: Focus on protein thiol oxidation and dysferlinopathies. FEBS J..

[51] Newman AB (2006). Strength, but not muscle mass, is associated with mortality in the health, aging and body composition study cohort. J. Gerontol. A Biol. Sci. Med. Sci..

[52] Li R, Xia J, Zhang X, Gathirua-Mwangi WG, Guo J, Li Y (2018). Associations of muscle mass and strength with all-cause mortality among US older adults. Med. Sci. Sports Exerc..

[53] Rijk JM, Roos PRKM, Deckx L, Van den Akker M, Buntinx F (2016). Prognostic value of handgrip strength in people aged 60 years and older: A systematic review and meta-analysis. Geriatr. Gerontol. Int..

[54] Leong, D. P*. et al.* Prognostic value of grip strength: Findings from the prospective urban rural epidemiology (PURE) study. *Lancet*. **386**(9990), 266–73 (2015). 10.1016/S0140-6736(14)62000-6 25982160

[55] Lino VTS, Rodrigues NCP, O’Dwyer G, Andrade MKDN, Mattos IE, Portela MC (2016). Handgrip strength and factors associated in poor elderly assisted at a primary care unit in Rio de Janeiro Brazil. PLoS ONE.

[56] Metter EJ, Talbot LA, Schrager M, Conwit R (2002). Skeletal muscle strength as a predictor of all-cause mortality in healthy men. J. Gerontol. A Biol. Sci. Med. Sci..

[57] Taniguchi Y (2016). Prospective study of trajectories of physical performance and mortality among community-dwelling older Japanese. J. Gerontol. A Biol. Sci. Med. Sci..

[58] Hill C, James RS, Cox VM, Seebacher F, Tallis J (2020). Age-related changes in isolated mouse skeletal muscle function are dependent on sex, muscle, and contractility mode. Am. J. Physiol. Regul. Integr. Comp. Physiol..

[59] Brown JC, Harhay MO, Harhay MN (2016). Sarcopenia and mortality among a population-based sample of community-dwelling older adults. J. Cachexia Sarcopenia Muscle..

[60] Silveira EC (2017). SAVPEA: Association of bone mineral density with frailty, pre-frailty, and osteoporosis in community-dwelling elderly: A prospective study. J. Geriatr. Med. Gerontol..

[61] Silveira EA, da Ferreira CC (2017). Total and central obesity in elderly associated with a marker of undernutrition in early life—sitting height-to-stature ratio: A nutritional paradox. Am. J. Hum. Biol..

[62] Silveira EA, Vieira LL, de Souza JD (2018). Elevada prevalência de obesidade abdominal em idosos e associação com diabetes, hipertensão e doenças respiratórias. Cienc e Saude Coletiva..

[63] Pagotto VI, Ferreira dos Santos KI, Gomes Malaquias SI, Márcia Bachion MI, Aparecida Silveira EI (2018). Calf circumference: clinical validated. Rev Bras Enferm..

[64] Silveira EA, Pagotto V, Barbosa LS, de Oliveira C, Das Graças Pena G, Velasquez-Melendez G (2020). Accuracy of BMI and waist circumference cut-off points to predict obesity in older adults. Cienc e Saude Coletiva..

[65] Silveira EA, Ferreira CC, Pagotto V, Santos AS, Velasquez-Melendez G (2017). Total and central obesity in elderly associated with a marker of undernutrition in early life—sitting height-to-stature ratio: A nutritional paradox. Am. J. Hum. Biol..

[66] Pagotto V, Silveira EA (2014). Applicability and agreement of different diagnostic criteria for sarcopenia estimation in the elderly. Arch. Gerontol. Geriatr..

[67] World Medical A (2013). World Medical Association Declaration of Helsinki: Ethical principles for medical research involving human subjects. JAMA.

[68] Madden AM, Smith S (2016). Body composition and morphological assessment of nutritional status in adults: A review of anthropometric variables. J. Hum. Nutr. Diet..

[69] Habicht JP (1974). Estandarización de metodos epidemiológicos cuantitativos sobre el terreno. Bol la Of Sanit Panam..

[70] WHO Consultation on Obesity (1999: Geneva, Switzerland) & World Health Organization. Obesity : preventing and managing the global epidemic : report of a WHO consultation. World Health Organization. https://apps.who.int/iris/handle/10665/42330 (2000). 11234459

[71] SBD - Sociedade Brasileira de Diabetes. *Diretrizes da Sociedade Brasileira de Diabetes:* 2017–2018 (Clannad, 2017).

[72] Malachias MVB.et al. 7ª Diretriz Brasileira de Hipertensão Arterial. *Arq Bras Cardiol* **107**(3Supl.3), 1–83 (2016). 10.5935/abc.20160140 PMC5319461 27819379

