# Supplementary material for: Sarcopenia and mortality risk in community-dwelling Brazilian older adults
Source: Sci Rep. 2022 Oct 20;12:17531. doi: 10.1038/s41598-022-22153-9 (PMC9585028; doi:10.1038/s41598-022-22153-9)
Supplement: Supplementary file 1 — Supplementary Information. [file 41598_2022_22153_MOESM1_ESM.docx]

**Supplementary Table** [**1**](https://www.nature.com/articles/s41598-021-01581-z#MOESM3)**.** Multicollinearity analysis.

| **Variables** | **VIF** | **1/VIF** |
| --- | --- | --- |
| **EWGSOP1^a^** | 8.18 | 0.12 |
| **EWGSOP1^b^** | 6.77 | 0.15 |
| **EWGSOP1^c^** | 2.12 | 0.47 |
| **EWGSOP1^d^** | 1.45 | 0.69 |
| **Mean VIF** | 4.63 |  |

**Notes:** VIF: variance inflation factor

EWGSOP1^a^: Low Calf circumference + Low Handgrip strength.

EWGSOP1^b^: Low Skeletal muscle mass index + Low Handgrip strength.

EWGSOP2^c^: Low Handgrip strength + Low Calf circumference.

EWGSOP2^d^: Low Handgrip strength + Low Skeletal muscle mass index.

**Supplementary Table 2.** Tertiles of calf circumference (CC) according to body mass index (BMI).

| **Tertiles of CC** | **BMI** | | | | **p** |
| --- | --- | --- | --- | --- | --- |
|  | **≤18,49** | **18,5-24,99** | **25-29,99** | **≥30** |  |
| **1 (n=44)** | 5 (100) | 32 (62.7) | 7 (15.9) | 0 (0.0) | **<0.001** |
| **2 (n=44)** | 0 (0.0) | 14 (33.3) | 19 (43.2) | 8 (25.0) |  |
| **3 (n=44)** | 0 (0.0) | 2 (3.9) | 18 (40.9) | 24 (75.0) |  |

**Notes:** BMI: Body Mass Index; CC: calf circumference.

Statistical analysis:

Fisher's exact test.
